# Extent of posterolateral tibial plateau impaction fracture correlates with anterolateral complex injury and has an impact on functional outcome after ACL reconstruction

**DOI:** 10.1007/s00167-022-07282-y

**Published:** 2022-12-16

**Authors:** Andreas Flury, Sandro Hodel, Octavian Andronic, Dominik Kaiser, Benjamin Fritz, Florian B. Imhoff, Sandro F. Fucentese

**Affiliations:** 1 Orthopaedic Department, Balgrist University Hospital, University of Zurich, Forchstrasse 340, 8008 Zurich, Switzerland; 2 Department of Radiology, Balgrist University Hospital, University of Zurich, Forchstrasse 340, 8008 Zurich, Switzerland

**Keywords:** Anterior cruciate ligament, Bone bruise, Anterolateral complex, Pivot-shift, Tibial impaction fractures

## Abstract

**Purpose:**

The impact of posterolateral tibial plateau impaction fractures (TPIF) on posttraumatic knee stability in the setting of primary anterior cruciate ligament (ACL) tear is unknown. The main objective was to determine whether increased bone loss of the posterolateral tibial plateau is associated with residual rotational instability and impaired functional outcome after ACL reconstruction.

**Methods:**

A cohort was identified in a prospective enrolled study of patients suffering acute ACL injury who underwent preoperative standard radiographic diagnostics and clinical evaluation. Patients were included when scheduled for isolated single-bundle hamstring autograft ACL reconstruction. Exclusion criteria were concurrent anterolateral complex (ALC) reconstruction (anterolateral tenodesis), previous surgery or symptoms in the affected knee, partial ACL tear, multi-ligament injury with an indication for additional surgical intervention, and extensive cartilage wear. On MRI, bony (TPIF, tibial plateau, and femoral condyle morphology) and ligament status (ALC, concomitant collateral ligament, and meniscus injuries) were assessed by a musculoskeletal radiologist. Clinical evaluation consisted of KT-1000, pivot-shift, and Lachman testing, as well as Tegner activity and IKDC scores.

**Results:**

Fifty-eight patients were included with a minimum follow-up of 12 months. TPIF was identified in 85% of ACL injuries (*n* = 49). The ALC was found to be injured in 31 of 58 (53.4%) cases. Pearson analysis showed a positive correlation between TPIF and the degree of concomitant ALC injury (*p* < 0.001). Multiple regression analysis revealed an increased association of high-grade TPIF with increased lateral tibial convexity (*p* = 0.010). The high-grade TPIF group showed worse postoperative Tegner scores 12 months postoperatively (*p* = 0.035).

**Conclusion:**

Higher degrees of TPIFs are suggestive of a combined ACL/ALC injury. Moreover, patients with increased posterolateral tibial plateau bone loss showed lower Tegner activity scores 12 months after ACL reconstruction.

**Level of evidence:**

III.

## Introduction

Non-contact anterior cruciate ligament (ACL) injury results from a pivot-shift like mechanism [37], simultaneously damaging the anterolateral complex (ALC) in 50–80% of cases [1, 5, 36]. Accordingly, injury to the ALC has been identified as the most important risk factor for a high-grade pivot-shift in ACL-deficient knees [4, 7]. For this reason, recent studies recommend a combined ACL/ALC reconstruction (with anterolateral tenodesis) in patients with higher preoperative pivot-shift grades [9, 33].

In addition, due to anterior translation of the tibia on the femur and internal tibial rotation, trabecular bone marrow oedema is observed in up to 80% of patients with a complete ACL injury [28, 31]. The severity of bone contusions depends on the amount of energy imparted [17, 18]. A recently published study distinguished between bone marrow oedema and impaction fractures of the posterior aspect of the lateral tibial plateau, which result from an injury of even greater severity [1]. Such displaced impaction fractures were reported with a high prevalence in the setting of ACL tears in more than 50% of cases [1].

However, it is unclear whether posterolateral tibial plateau bone loss affects the outcome of ACL reconstruction, especially when the ALC is not simultaneously addressed.

The purpose of this study was to examine the influence of morphologic variants of tibial plateau impaction fractures (TPIF) on clinical parameters before and after ACL reconstruction, without addressing the ALC. It was hypothesised that increased posterolateral tibial plateau bone loss on MRI may be associated with higher grades of preoperative and postoperative rotational instability, and impaired 1-year functional outcome.

## Materials and methods

This is a secondary analysis of prospectively collected data from a randomised-controlled study approved by the Institutional Review Board and the local ethical committee (Zurich Cantonal Ethics Commission, 2017-00750).

### Study population

**Fig. 1:**
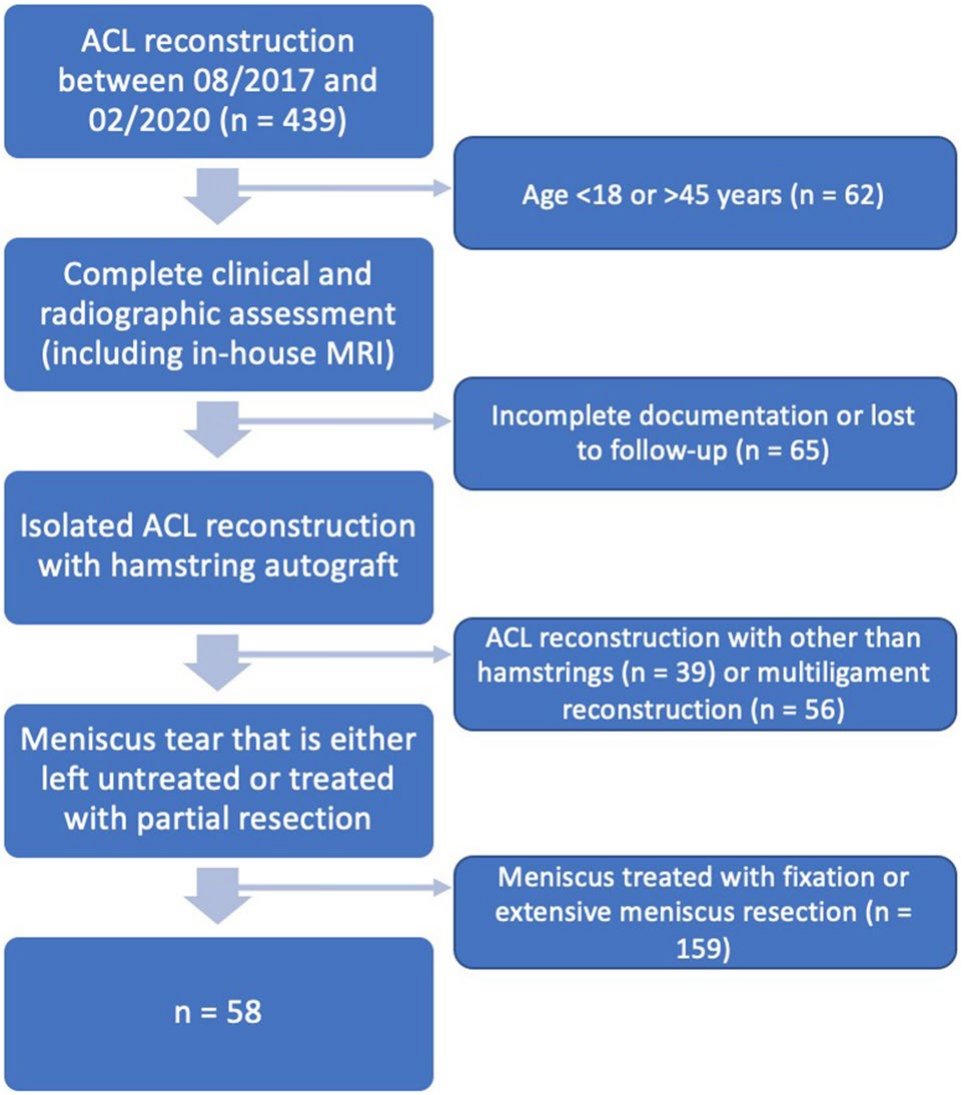
Flowchart of patients’ recruitment

Inclusion criteria were: isolated single-bundle hamstring autograft ACL reconstruction within 8 weeks of contact or non-contact ACL injury, standard in-house MRI assessment, clinical evaluation by the senior surgeon under anaesthesia, and a completed 1-year follow-up. Exclusion criteria were combined ACL/ALC reconstruction, age < 18 or > 45 years, previous surgery or symptoms in the affected knee, partial ACL tear, previous or current ACL injury on the contra-lateral leg, multi-ligament damage with an indication for additional surgical intervention, extensive cartilage damage (Outerbridge > III), clinically excessive varus/valgus leg axis, and meniscus tears treated with fixation and, therefore, interfering with the rehabilitation protocol (Fig. 1). Diagnosis of meniscal tears as well as accessibility and necessity of fixation required arthroscopic confirmation. Patients who underwent partial meniscectomy were included in the study.

Finally, 58 patients treated for an acute ACL lesion between 2017 and 2020 were prospectively enrolled and followed up for a minimum of 12 months after ACL reconstruction.

### Surgical technique and rehabilitation protocol

The semitendinosus and (in case of a graft diameter < 8 mm) gracilis tendons were harvested through a small incision over the pes anserinus. All femoral tunnels were drilled through an anteromedial portal with anatomic ACL graft position and orientation. The tibial tunnel was drilled in 45° of flexion. The grafts were secured using a suspensory device (Karl Storz; Fliptack femoral, Endotack tibial). At the tibial side, an additional 23 mm long bioresorbable interference screw (Mega fix, Karl Storz) was used (hybrid fixation technique).

The knee was first immobilised in an extended position for 5 to 7 days with immediate postoperative active and passive range of motion exercises. For a total of 6 weeks, all patients used a hinged knee brace with partial weight-bearing starting from the first week, with full weight-bearing at 3 weeks. Jogging and running were allowed 3 months postoperatively. Return to athletic sports with pivoting elements was allowed not earlier than 9 months after ACL reconstruction.

### Clinical evaluation

Demographic (body mass index (BMI), age, sex) and clinical information (mechanism of injury, preoperative and 1-year postoperative Tegner activity score, and IKDC scores) were recorded for all patients, as well as data from operative reports (concomitant meniscal injury, performed meniscal procedures).

Physical examination consisted of KT-1000 measurements, Lachman test, and the pivot-shift. The pivot-shift test was performed under anaesthesia by the senior author before surgery and was graded according to the classification of the International Knee Documentation Committee (IKDC): grade 0 (normal), grade 1 (glide), grade 2 (clunk), or grade 3 (locked subluxation) [11].

### Radiographic assessment

**Fig. 2:**
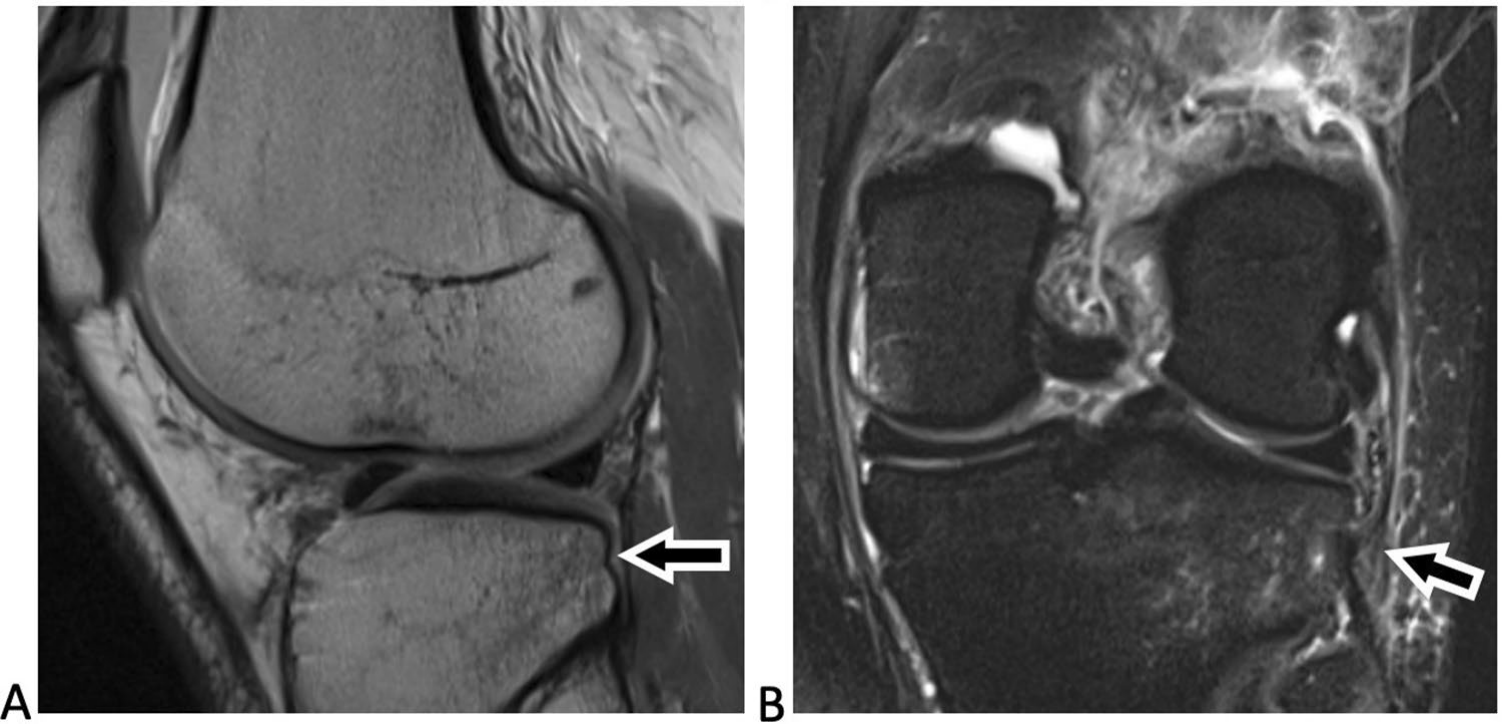
Magnetic resonance imaging of the left knee joint of a 24-year-old patient after ACL injury. The sagittal proton-density weighted image (**A**) shows a posterolateral tibial plateau fracture not involving the articular surface with a small cortical buckle (arrow in **A**), corresponding to a Type I fracture. The coronal T2-weighted fat-suppressed image (**B**) shows hyperintense signal alteration with intact fibres of the distal anterolateral complex (arrow in B), consistent with a low-grade injury

**Fig. 3:**
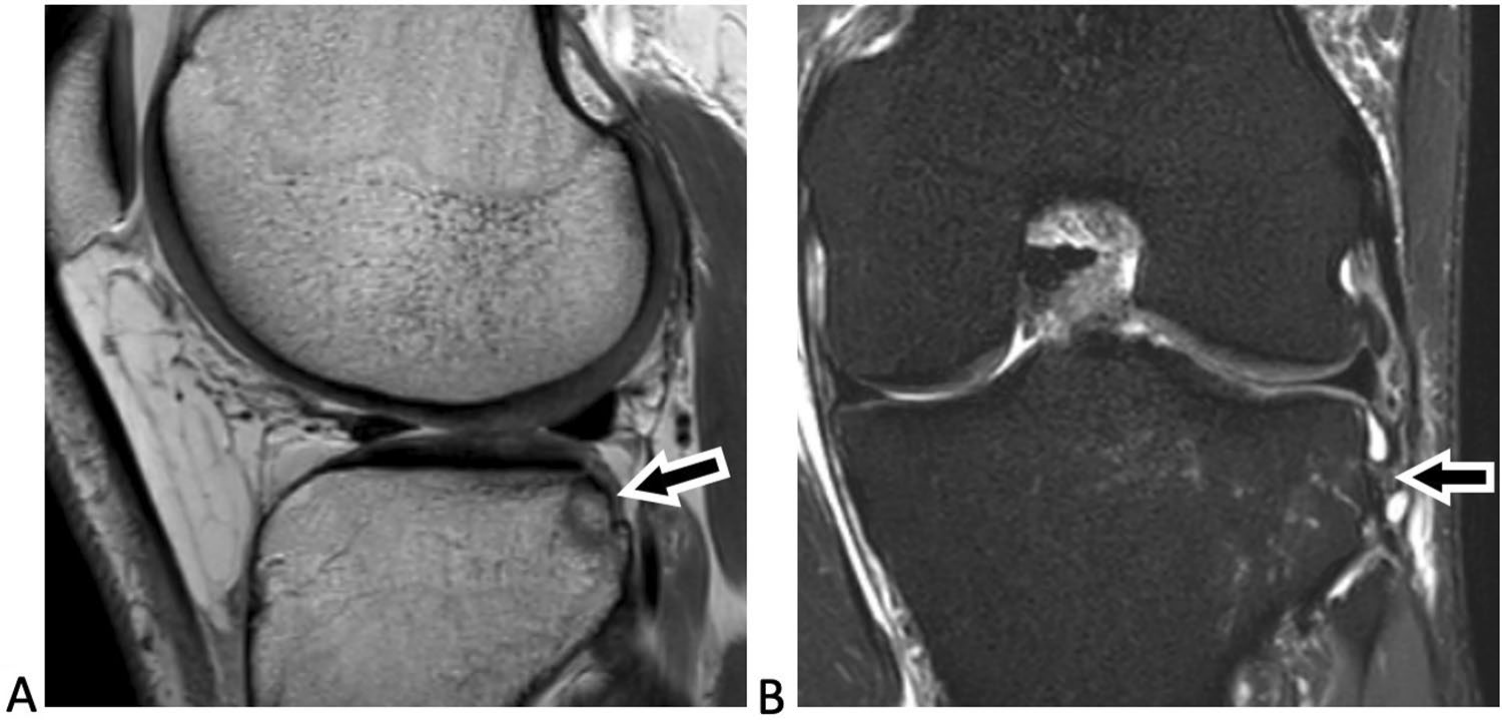
Magnetic resonance imaging of the left knee joint of a 43-year-old patient with an acute ACL injury. The sagittal proton-density-weighted image (**A**) shows a posterolateral tibial plateau fracture with an impacted fragment (arrow in **A**), corresponding to a Type IIIb fracture. The coronal T2-weighted fat-suppressed image (**B**) shows hyperintense signal alteration with partial tearing of the distal fibres of the anterolateral complex (arrow in **B**), consistent with a high-grade injury

Preoperative MRI scans were analysed on a picture archiving and communicating system (PACS) workstation and reviewed for the presence of displaced posterolateral TPIF by two observers (one senior orthopaedic resident and one fellowship-trained musculoskeletal radiologist), blinded to clinical data. MRI signal change at the posterolateral tibial plateau was classified as impaction fracture only if there was a displacement of subchondral or cortical bone at the posterolateral tibial plateau rim visible on sagittal MRI T1-weighted images, according to Bernholt et al. [1]. TPIFs were graded into type I fractures when the articular surface was not involved (Fig. 2), type IIA with articular surface involvement and bone loss of less than 10% of the tibial plateau, and type IIB with more than 10% bone loss [1]. Type III fractures were defined as impaction fractures resulting in a displaced bony fragment, subclassified into type IIIA with a shear fragment and type IIIB with a depressed fragment (Fig. 3) [1]. For this study, type 0 and I TPIF were classified as low-grade, and type II or above as high-grade.

In the event of discrepancies concerning the morphologic variant of TPIF, the case was discussed among the co-authors to reach consensus. The MRI of all cases were reviewed again after 6 weeks, leading to an adjustment of the classification in one case.

The injury severity of the anterolateral complex (ALC) was defined in terms of discontinuity of anterolateral ligament fibres: complete (grade 3) or partial (grade 2) disruption. If only periligamentous oedema existed with identifiable, continuous low signal intensity fibres, the ALC was considered intact and either classified as strained (grade 1) or unharmed (grade 0) [36]. Frontal anteroposterior radiographs were evaluated for the Segond fracture according to established criteria (a visible bone flake at the superolateral rim of tibia) [6].

All patients were examined on clinical 1.5 Tesla or 3 Tesla MR scanners. All included MRI consisted of fluid sensitive fat-suppressed and non-fat-suppressed MR sequences in three orthogonal planes, including sagittal and axial cartilage sensitive sequences.

Following bony morphological parameters of the tibial plateau and the femoral condyle were analysed on MR scans due to their previously described correlation with an increased pivot-shift [12, 26, 27, 35]: lateral and medial tibial plateau depth (convexity/concavity) [21, 24], medial/lateral posterior tibial slope [3, 16, 19], as well as the lateral femoral condyle index (LCFI) which is a risk factor for ACL injury [13]. Measurement (accuracy per pixel: 0.1 mm and 0.1°) was performed in a standardised technique and outcome variables are given by one decimal.

### Statistical analysis

Means and standard deviations were used for descriptive analysis for continuous variables (independent *t* test) and frequencies or percentages for discrete or dichotomous variables (Chi-squared or Fisher’s exact test). For further analysis, TPIFs were classified into low-grade (0 and 1) or high-grade (2a or greater) and included as a dichotomous variable. Grade 0 also included isolated bone bruise without TPIF. A paired Wilcoxon test was used to determine statistical differences between baseline values among groups. For determination of correlations, a bivariate Pearson analysis was used to search for injury and instability factors associated with a high-grade posterolateral TPIF and namely: associated meniscal tears, injuries of collateral ligaments, concomitant Segond fractures or ALC injuries, as well as intraoperative pivot-shift testing.

In order to look for demographic (age, gender, BMI) or bony morphological factors (LFCI, medial and lateral tibial slope, delta slope, and tibial plateau depth), that may predispose to high-grade TPIF, a linear multiple regression analysis was performed. An a-priori power analysis (fixed model) for a medium effect size (*f*^2^) = 0.15 and a desired statistical power of 1 − *β* > 0.8 (total number of predictors: 9) was performed in order to secure a sufficient sample size for the multiple regression analysis regarding demographic and bony morphological predictors for high-grade TPIF. The minimum sample size of *n* = 55 was, therefore, achieved with the current study population (*n* = 58).

A one-way analysis of variance (ANOVA) was performed to determine any associations of the degree of TPIF with postoperative clinical outcome: continuous variables (Tegner activity score, IKDC) and dichotomous (presence (grades 1–3) or absence (grade 0) of pivot-shift). An a-priori power analysis for ANOVA (fixed effects, omnibus, one-way) was performed for a medium effect size (*f*^2^) = 0.25 and a desired statistical power of 1 − *β* > 0.95 (total number of compared groups: 5). With *n* = 58, the minimum sample size for this analysis was achieved as well.

All statistical tests were 2-sided, and *p* value of < 0.05 was considered statistically significant. Statistical analysis was performed with SPSS (version 23.0; IBM SPSS Statistics) and G*Power (version for Mac 3.1.9.6).

## Results

Posterolateral TPIFs were identified in 49 of 58 acute ACL injury cases (84.5%), of which 20 (34.4%) were classified as type I, 21 (36.2%) as type IIa, seven (12.1%) as type IIb, and one (1.8%) as type IIIb. ALC injury was detected in 31 of 58 (53.4%) cases. Preoperative assessment indicated a high-grade (II and III) pivot-shift in 29% of patients (*n* = 17). All preoperative data are presented in Table 1, classified by either high or low-grade TPIF.

**Table 1:** Preoperative values of demographical, clinical, and radiographic data of patients, classified by the grade of the posterolateral tibial plateau impaction fracture

Variable	Low-grade TPIF group (*n* = 29)	High-grade TPIF group (*n* = 29)	*p* value
*Demographical data*			
Age (years)	26.5 ± 7.9	30.7 ± 7	0.039
BMI (kg/m^2^)	24.4 ± 3.4	24.3 ± 3	n.s.
Gender			n.s.
Male	19 (66%)	17 (59%)	
Female	10 (34%)	12 (41%)	
Mechanism of injury			n.s.
Contact	5 (17%)	2 (7%)	
Non-contact	24 (83%)	27 (93%)	
*Clinical data*			
Associated meniscus injury	13	9	n.s.
Associated MCL/LCL injury	1 (4%)/2 (7%)	4 (14%)/1 (4%)	n.s.
Side-to-side difference of KT-1000			n.s.
Preoperative	4 ± 1.8	3.5 ± 1.6	
Postoperative	0.4 ± 0.6	0.5 ± 0.7	
Pivot-shift, preoperative			n.s.
Low-grade (grade 0 and I)	18 (62%)	23 (79%)	
High-grade (grade II and III)	11 (38%)	6 (21%)	
Tegner score, preoperative	7 (3–9)	6 (4–10)	n.s.
Tegner score, postoperative	5 (3–9)	5 (1–9)	0.05
IKDC score, postoperative	86.2 ± 11.4	81.7 ± 11.2	n.s.
*Radiographical data*			
Medial tibial slope (°)	3.8 ± 2	3.7 ± 2.7	n.s.
Lateral tibial slope (°)	5.7 ± 3	5 ± 2.8	n.s.
Delta slope (°)	2 ± 2.5	1.3 ± 2	n.s.
Medial tibial plateau depth (mm)	2.7 ± 0.7	2.6 ± 0.7	n.s.
Lateral tibial plateau depth (mm)	2 ± 0.8	2.6 ± 1.1	0.026
LFCI	0.6 ± 0	0.7 ± 0	n.s.
Grade of ALC injury			< 0.0001
None/strain	22 (76%)	5 (17%)	
Partial/complete rupture	7 (24%)	24 (83%)	
Segond fracture	0	3 (10%)	n.s.
Morphologic variant of TPIF			< 0.0001
Type 0	9 (31%)	0	
Type I	20 (69%)	0	
Type IIa	0	21 (72%)	
Type IIb	0	7 (24%)	
Type IIIa	0	0	
Type IIIb	0	1 (4%)	

Continuous variables are shown as mean ± standard deviation, categorical variables are shown as number of patients and percentage of the group, and Tegner score is given with median and range values

*BMI* body mass index, *MCL* medial collateral ligament, *LCL* lateral collateral ligament, *LFCI* lateral femoral condyle index, *ALC* anterolateral complex, *TPIF* tibial plateau impaction fracture

Pearson analysis showed a positive correlation between high-grade posterolateral TPIF and concomitant ALC injuries, regardless of whether analysed as dichotomous and/or continuous variables (*p* < 0.001). There was, however, no correlation with other associated lesions or the intraoperative testing of instability: meniscal tears, collateral ligament injury MCL/LCL, Segond fracture, and grade of pivot-shift (all n.s.).

Multiple regression analysis of predisposing demographic and morphologic factors revealed an increased association of high-grade TPIF with increasing age (*p* = 0.036), as well as with increased lateral tibial convexity (*p* = 0.010) (Table 2).

**Table 2:** Multiple regression analysis (*R*^2^ = 0.236) on predisposing demographic and morphologic factors associated with high-grade posterolateral tibial plateau fractures

Predisposing factor	Totals or average value (SD or range)	Significance	Odd’s ratio
Age (years)	28.6 ± 7.7	**0.036**	1.1
Gender	36 males, 22 females	0.357	
BMI (kg/m^2^)	24.3 ± 3.2	0.769	
LFCI	0.6 (0.5–0.8)	0.808	
Medial tibial slope (mm)	3.7	0.514	
Lateral tibial slope (mm)	5.3	0.211	
Delta slope (mm)	1.6	0.218	
Medial tibial plateau depth (mm)	2.6	0.362	
Lateral tibial plateau depth (mm)	2.3	**0.01**	2.8

Bold = statistically significant values

*BMI* body mass index, *LFCI* lateral femoral condyle index

There was no significant difference among groups at the baseline in terms of Tegner activity score, as determined by the paired Wilcoxon test (n.s.). Looking at the Tegner score 12 months postoperatively, there was a statistically significant worse activity in the high-grade TPIF group (*p* = 0.05). A residual postoperative pivot-shift phenomenon (grade 1) was present in two patients (6.9%), of which both had a high-grade TPIF (n.s.).

No ACL re-rupture occurred in this study cohort. Nevertheless, there were three (10%) reoperations in the high-grade TPIF group versus one (3%) in the low-grade group (n.s.), on average 9.75 months after index surgery. Reoperation was performed due to meniscus injury (*n* = 2) or limited range of motion because of intraarticular scarring (*n* = 1). One patient with a preoperative grade 2a TPIF experienced a further collapse of the posterolateral tibial plateau 9 months postoperatively. Arthroscopic-assisted reduction was performed and the void was filled with cancellous allograft. At the 2-year follow-up, the patient achieved an IKDC score of 83.9 points.

## Discussion

The most important finding of this study was that a relationship exists between posterolateral tibial plateau bone loss and incidence of concomitant ALC injuries. Moreover, patients with increased posterolateral tibial plateau bone loss had a lower Tegner activity score 12 months postoperatively.

Several studies confirmed the contributing role of the ALC in controlling excessive tibial internal rotation in the absence of the ACL [30, 33]. In biomechanical studies, an increased rotational instability could be shown in all degrees of flexion after cutting the ALC in an ACL-deficient knee [26, 34]. A correlation between ALC injury and pivot-shift grade was described in two recent clinical studies [23, 32]. However, the authors of the two studies reported inconsistent results regarding the relationship between bone bruise and ALC integrity after ACL injury. Impaction fractures of the posterolateral tibial plateau result from an injury of even greater severity compared to bone bruise alone. Morphologic variants of TPIF were recently described by Bernholt et al. [1]. However, it is unknown how bone loss of the posterolateral tibial plateau should be interpreted and/or managed while addressing the ACL injury. In a recent metanalysis, Filardo et al. [8] reported that the presence of subchondral fractures and the lateral location of bone bruise correlated with a higher instability and range of motion limitation, and might, therefore, negatively influence the clinical outcome and return to full activity after ACL reconstruction.

The present study, based on a patient cohort investigated before and after ACL without ALC reconstruction, demonstrates the following: first, there is a positive correlation between high-grade posterolateral TPIF and the incidence of concomitant ALC injuries (*p* < 0.001). Second, the convexity of the lateral tibia plateau is associated with higher graded TPIF. According to Viskontas et al. [37], non-contact ACL injuries result from an anterior translation of the tibia on the femur and internal tibial rotation and cause more severe bone bruising. The contact mechanism predominantly happens due to a valgus force in combination with translation [37]. The combined injury pattern of ALC/ALC with higher-grade TPIF is most likely explained by the extent of the pivoting mechanism, even resulting in increased preoperative tibial internal rotation [15]. Accordingly, the high-grade TPIF group included (insignificantly) more non-contact and less contact ACL injuries compared to the low-grade TPIF group. Moreover, according to the tibia morphology, some knees are probably more susceptible to pivoting than others [14]. However, it is suggestive that excessive pivoting force, while resulting in higher graded TPIF and ALC injury, should also cause increased assessable instability. Nevertheless, no correlation between preoperative pivot-shift and TPIF or ALC injury was found. The pivot-shift test is very examiner-dependent, which was shown by the fact that the degree of the pivot-shift inversely correlated with the size of the knee, measured by the extension and flexion curvature of the femoral condyle, as used for the LCFI [13]. Several previous studies confirmed a correlation between the grade of preoperative pivot-shift and ALC injury [7, 23, 32]. Therefore, this inconsistency should not be weighted too much.

Regarding the functional outcome 12 months after surgery, Tegner scores of the high-grade TPIFs were significantly lower than in the low-grade group (*p* = 0.04). Compared to the literature, the high-grade TPIF group’s score was even lower compared to 2274 primary ACL reconstructions of the Danish ACL reconstruction registry [22]. This is in accordance with a recently published study by Bernholt et al., confirming inferior postoperative outcomes in high-grade TPIFs 2 years after surgery [2].

Nevertheless, this study is not able to define whether the impaired functional outcome results from residual rotational instability. Even if so, it does not allow any statement to be made about which structure mainly causes the residual rotational instability: osseous (TPIF) or ligamentous (ALC) disintegrity. or a combination of these two. While it is well known that ALC injury, geometry of the lateral tibia plateau [13], and posterior root tears of the lateral meniscus [10] all increase rotatory instability, the effect of posterolateral tibial bone loss in ACL-intact and ACL-deficient knees needs further investigation. Accordingly, indication for treatment of these fractures is still under debate. From the biomechanical aspect, the lack of more than 10% of the posterolateral tibia or depression of more than 2 mm might result in increased rotational instability of the ACL-deficient knee. Previous studies that treated TPIF-graded 2b or higher with fracture fixation reported good clinical outcomes [20, 25]. The number of here reported and higher graded TPIFs is too small to draw reasonable conclusions in this regard. In this cohort, only one patient experienced further collapse of a type 2a TPIF, which is why further investigation is needed. The correct algorithm for treating these cases is, therefore, still unclear. This study’s relevance, however, lies in that high-grade TPIF should raise awareness of potentially increased rotational instability after ACL reconstruction and, therefore, trigger concurrent ALC reconstruction or TPIF fracture fixation [29].

Strengths of this study include the prospective design with excellent documentation of a large amount of clinical and radiographic data. However, the study should be interpreted in light of its potential limitations. First, 12 months is a short follow-up time, allowing only a restricted statement about rates of return to sport or graft failure. However, the goal was to primarily investigate the clinical impact of posterolateral bone loss related to ACL injury, whose clinical relevance could be clearly demonstrated herewith. Next, MRI interpretation of TPIF and status of ALC status could be criticised, as the most reliable modality for diagnosing an ALC injury is subject to ongoing debates [4]. However, all images were reviewed several times by orthopaedic surgeons and a musculoskeletal radiologist, and a consensus was reached in all cases after consultation among co-authors. Therefore, a high degree of accuracy and reliability can be assumed. Finally, some degree of selection bias is suggestive because meniscus tears amenable to fixation were excluded due to the selection criteria of the principle study. Moreover, multi-ligament injuries were excluded as well, although it can be assumed that higher energy is applied to the knee in these cases. Clinical assessment, however, would be less conclusive if multi-ligamentous injuries had also been included.

## Conclusion

Increased posterolateral tibial plateau bone loss is suggestive of a combined ACL/ALC injury. Moreover, patients with increased posterolateral tibial plateau bone loss had a lower Tegner activity score 12 months after ACL reconstruction, which is indicative of increased rotational instability after ACL reconstruction.
